# Expression of PSMD14 in lung adenocarcinoma and its impact on immune cell infiltration and prognosis: a comprehensive analysis based on RNA and single-cell RNA sequencing

**DOI:** 10.3389/fimmu.2025.1560693

**Published:** 2025-05-22

**Authors:** Jing Zhang, Bohao Sun, Jiabin Lai, Weike Kong, Nan Wang, Panyuan Li, Yichen Wu, Zhaochang Jiang

**Affiliations:** ^1^ Department of Pathology, Second Affiliated Hospital, School of Medicine, Zhejiang University, Hangzhou, Zhejiang, China; ^2^ Department of Pharmacy, Zhejiang Provincial People’s Hospital, People’s Hospital of Hangzhou Medical College, Hangzhou, China; ^3^ Department of Pathology, Sir Run Run Shaw Hospital, Zhejiang University School of Medicine, Hangzhou, China

**Keywords:** LUAD, PSMD14, poor-prognosis predictor, clinical stage, single cell sequencing

## Abstract

**Background:**

Lung adenocarcinoma (LUAD) is distinguished by intricate relationships between tumor advancement and the immune microenvironment. The function of PSMD14 (Proteasome 26S Subunit, Non-ATPase 14) within the context of LUAD is not well elucidated, especially in terms of its correlation with immune cell infiltration and the prognosis of patients.

**Methods:**

The objective of this research was to explore the expression levels of PSMD14 in LUAD and to evaluate its potential implications for tumor immunity and clinical outcomes. A multifaceted approach was adopted, which included the analysis of RNA sequencing (RNA-seq) data, assessment of immune cell infiltration, survival analysis, gene enrichment analysis, and integration of single-cell RNA-seq data to thoroughly evaluate the biological relevance of PSMD14. Furthermore, we examined the correlation between PSMD14 expression and clinical parameters. Immunohistochemistry techniques were employed to analyze PSMD14 expression in samples of invasive pulmonary adenocarcinoma.

**Results:**

Our study demonstrated that the expression of PSMD14 is markedly elevated in LUAD and exhibits a positive correlation with other members of the JAMM family, including EIF3H and PSMD7. Importantly, elevated levels of PSMD14 were linked to poor patient prognosis, indicating its potential utility as a biomarker. Moreover, Kyoto Encyclopedia of Genes and Genomes (KEGG) pathway analysis revealed that PSMD14 is significantly associated with pathways related to the cell cycle and nicotine dependence, underscoring its vital function in modulating cell proliferation and metabolic activities. Furthermore, PSMD14 expression was found to be associated with the infiltration of immune cells, particularly influencing T helper and Th2 cell populations, and exhibited an inverse relationship with several immune checkpoint molecules, such as PD-1 and TIGIT. Insights from single-cell RNA sequencing identified that PSMD14-expressing immune cell types in LUAD include dendritic cell (DC), monocytes, and tissue stem cells. These findings highlight the role of PSMD14 in the immune evasion strategies prevalent in LUAD. Additionally, a notable increase in PSMD14 protein levels was recorded in LUAD patients, with expression levels correlating with tumor size, lymph node involvement, and the TNM classification.

**Conclusion:**

In summary, our research underscores the crucial role of PSMD14 in LUAD, highlighting its promise as a potential target for therapy and a prognostic indicator. Furthermore, it opens up novel approaches for future therapeutic interventions.

## Introduction

Lung adenocarcinoma (LUAD) poses a significant global health issue, distinguished by its aggressive behavior and unfavorable prognosis (1, 2). This malignancy not only places a heavy burden on affected individuals but also incurs substantial economic costs for healthcare systems across the globe. The challenge of early diagnosis of lung adenocarcinoma arises from its multifaceted etiology and the propensity for early distant metastasis at early stages, which frequently manifests as the first symptom recognized by healthcare professionals or patients themselves (3, 4). The pathogenesis of lung adenocarcinoma is driven by complex regulatory networks, underscoring the intricacies of its developmental processes. Unfortunately, the prognosis for patients at advanced stages remains grim due to extensive cancer spread, emphasizing the urgent necessity for early detection, diagnosis, and treatment strategies for this carcinoma (5, 6). Current therapeutic approaches, encompassing surgical interventions, chemotherapy, and targeted treatments, have demonstrated limited effectiveness, particularly in the later stages of the disease (7). Despite significant progress in the comprehension of tumor biology, a crucial gap persists in clarifying the contributions of specific molecular entities, such as PSMD14, within the tumor immune microenvironment and their relevance to patient prognoses (8, 9). Although prior investigations have recognized various mechanisms of immune evasion, the exact correlation between PSMD14 expression, tumor immune infiltration, and patient outcomes in LUAD has not been comprehensively explored. Consequently, this research is vital to fill this knowledge gap, potentially facilitating the discovery of new biomarkers and therapeutic targets that may improve the management of LUAD.

The tumor microenvironment (TME) is integral to the progression and immune modulation of diverse cancers, especially LUAD (10, 11). This intricate and evolving ecosystem encompasses cancerous cells, stromal components, extracellular matrix constituents, and a variety of immune cell types (12, 13). The TME significantly influences tumor proliferation, metastasis, and treatment responses. Recent investigations underscore the intricate relationships between tumor cells and immune cells within the TME, illuminating how tumors can circumvent immune surveillance through various immune evasion strategies. A comprehensive assessment of these immune evasion tactics can be achieved through multi-omics analyses, allowing for prognostic predictions utilizing the immune inflammation index. For example, the integrated machine learning and genetic algorithm-driven multi-omics analysis (iMLGAM) has proven effective in forecasting the responses of several cancers to immune checkpoint inhibitors (ICB) (14). A reduced iMLGAM score correlates with beneficial immune activation patterns, whereas an elevated score suggests the presence of immune evasion mechanisms. Furthermore, the systemic immune inflammation index (SII), derived from platelet, neutrophil, and lymphocyte counts, has been established as a prognostic predictor for various solid tumors (15). In Zhang et al.’s study, PTMLS is considered a valuable tool for assessing personalized prognosis and stratification of immunotherapy in LUAD (16). Its inhibition may enhance the effectiveness of immunotherapy and could serve as a new target for LUAD management. This investigation establishes a foundation for biomarker-driven precision oncology strategies. Nonetheless, additional research is warranted to explore the interactions between PSMD14 and the TME and their implications for immune evasion in LUAD. Gaining insight into these interactions is vital for the development of targeted therapies capable of effectively modulating the TME to bolster anti-tumor immunity.

PSMD14, an integral component of the 26S proteasome, is pivotal in the ATP-dependent degradation of ubiquitinated proteins, representing a multi-protein complex that is crucial for maintaining cellular protein equilibrium (17–19). This protein facilitates the deubiquitination of proteins within the cell, a process that is essential for their subsequent degradation. Such a mechanism is critical for the elimination of misfolded or damaged proteins that may disrupt cellular operations, as well as the removal of proteins that are no longer required by the cell (17). Ubiquitination, a vital post-translational modification, significantly influences processes such as cell proliferation, apoptosis, and tumor development. Consequently, a thorough examination of the ubiquitination pathway and its critical enzymes in lung adenocarcinoma is of considerable clinical relevance. PSMD14 is classified within the JAMM/MPN+ metallopeptidase family and possesses a JAMM domain; its deubiquitinase function is reliant on Zn²^+^ (20). By cleaving ubiquitin chains from target proteins, PSMD14 promotes their further degradation via the proteasome (19, 21). Conditions linked to PSMD14 include glioma, multiple myeloma, and breast cancer, underscoring its characterization as an oncogene (18). In contrast to earlier investigations concerning PSMD14 in LUAD (10, 22), our study offers a comprehensive examination of PSMD14 utilizing pan-cancer analysis, functional enrichment assessment, immune infiltration evaluation, JAMM family correlation assessment, and prognostic analysis. Notably, we gathered clinical data from patients with lung cancer to validate the association between PSMD14 and various prognostic clinical indicators. Investigating the role and mechanisms of PSMD14 in these types of cancer could yield transformative insights into their underlying pathogenesis and reveal potential therapeutic targets, thereby creating new possibilities for treatment strategies against these challenging diseases.

The current investigation focuses on the expression levels of PSMD14 in LUAD and its complex association with the tumor immune microenvironment, as well as its implications for patient prognosis. Prior studies have identified links between various immune-related genes and the advancement of tumors; nevertheless, the distinct role of PSMD14 in immune cell infiltration and its prognostic significance has not been thoroughly examined. This identified gap in existing research highlights the innovative aspect of our study, where we strive to clarify the potential of PSMD14 as a biomarker for LUAD. Our results reveal that increased expression of PSMD14 is associated with poorer clinical outcomes, indicating its potential role in tumor progression and mechanisms of immune evasion.

This investigation utilizes a comprehensive research strategy that incorporates RNA sequencing (RNA-seq) data analysis, immune infiltration evaluation, survival analysis, gene enrichment analysis, and the integration and analysis of single-cell RNA-seq data. The strength of this multifaceted approach lies in its capacity to clarify the expression patterns and biological significance of PSMD14 from multiple dimensions, thus offering a complete perspective on its function in LUAD (23, 24). Notably, the study underscores the critical role of single-cell RNA sequencing (scRNA-seq) technology in oncological research. In contrast to conventional transcriptome sequencing techniques, scRNA-seq delivers intricate, cell-specific information and uncovers cellular diversity within the tumor microenvironment (25, 26). The main aim of this research is to examine the expression patterns of PSMD14 in LUAD, investigate its association with immune infiltration, and evaluate its influence on patient outcomes. By addressing these pivotal elements, this study aspires to provide significant insights into the potential of PSMD14 as a promising biomarker for diagnostic and therapeutic strategies in lung adenocarcinoma, ultimately advancing our understanding of tumor biology and the immune microenvironment.

## Material and methods

### Gene expression profiles of tumor and adjacent normal tumor tissues

The RNA sequencing data for both normal and tumor samples were obtained from the The Cancer Genome Atlas (TCGA) (http://cancergenome.nih.gov) and Genotype-Tissue Expression (GTEx) (http://commonfund.nih.gov/GTEx/) initiatives. The validation cohorts, which included comprehensive expression profile data from GSE31210 was sourced from the GEO database (https://www.ncbi.nlm.nih.gov/gds). Furthermore, the single-cell RNA sequencing dataset identified as GSE117570 was sourced from the GEO database.

### Construction of the JAMM-associated risk model

Utilizing the TCGA database, a total of seven potential JAMM-associated genes were identified through the application of the least absolute shrinkage and selection operator (LASSO) Cox regression analyses. This approach was employed to minimize redundancy and mitigate the risk of model overfitting. Following this, three genes were chosen to construct a prognostic risk-scoring model aimed at forecasting overall survival (OS) in patients diagnosed with LUAD.

### Development of nomograms

Nomograms that integrate clinical characteristics and the PSMD14 models were formulated utilizing the “rms” R package (version 6.3) to forecast OS in LUAD, drawing upon data sourced from the TCGA cohort. To evaluate the predictive accuracy of these nomograms, a thorough assessment was performed using time-dependent calibration curves, which enabled the comparison of forecasted outcomes against actual survival data over time. Moreover, a univariate Cox regression analysis was conducted to ascertain whether the PSMD14 model could function as an independent prognostic indicator for OS in LUAD. Additionally, receiver operating characteristic (ROC) curves were employed to compute the area under the curve (AUC) value, which provided a quantitative evaluation of the diagnostic effectiveness and applicability of the nomogram in forecasting patient outcomes.

### Immune infiltration analysis

Utilizing the ssGSEA algorithm incorporated within the R package GSVA version 1.46.0 (27), we employed the immune cell markers delineated in the described in the pertinent immunological literature to assess the immune infiltration levels in the associated corresponding cloud data (28).

### Survival analysis

The prognostic data were derived from the study conducted by Liu et al. (29). We performed tests for the proportional hazards assumption assumptions along with Cox regression analysis utilizing the survival package, and subsequently created visual representations in the form of forest plots with ggplot2. The Kaplan-Meier (KM) analysis was executed using the survival package to assess the proportional hazards assumption assumptions and to fit survival regression models. Results were depicted using the survminer and ggplot2 packages for enhanced visualization. Furthermore, we assessed the prognostic significance of PSMD14 by analyzing clinical data obtained from the TCGA database. For statistical analysis and visualization, the “Survival” (version 3.2-10) and “survminer” (version 0.4.9) packages were employed, respectively.

### Gene enrichment analysis

Enrichment analyses utilizing Gene Ontology (GO) and the Kyoto Encyclopedia of Genes and Genomes (KEGG) were conducted on the differentially expressed genes associated with LUAD through the application of the R package clusterProfiler. The samples were stratified into high and low-expression groups based on the median expression level of the gene PSMD14. Gene Set Enrichment Analysis (GSEA) was executed using the clusterProfiler package (version 4.8.0) (22, 23). Significant results were defined as gene sets that exhibited a normalized enrichment score (NES) greater than 1, with a false discovery rate (FDR) of less than 0.05.

### Single-cell RNA−seq data integration and analysis

The single-cell RNA sequencing dataset identified as GSE117570 underwent processing through the Seurat package (version 4.0.5) within the R programming environment. A series of quality control measures were implemented to filter the cells, which included maintaining a mitochondrial UMI ratio of less than 10%. These protocols were essential for the elimination of substandard cells, thereby ensuring the creation of a reliable and comprehensive dataset for subsequent analyses. The normalized data were merged using the LogNormalization method. Following this, cell clustering was performed through the “FindNeighbors” and “FindClusters” functions (30, 31). To visualize the resulting cell clusters, PCA, tSNE, and UMAP techniques were employed. The annotations for cell types were derived by extracting cell cluster markers from the CellMarker 2.0 database (http://bio-bigdata.hrbmu.edu.cn/CellMarker/) (32, 33).

### Patients and tissue specimens

Tissue specimens from patients diagnosed with lung adenocarcinoma, glioma, buccal mucosa carcinoma, gingival carcinoma, tongue carcinoma, liver cancer, as well as adjacent non-cancerous tissues, were sourced from the Department of Pathology at the Second Affiliated Hospital, Zhejiang University School of Medicine. This investigation strictly followed the ethical guidelines outlined in the Declaration of Helsinki. Exclusion criteria encompassed: i) the presence of autoimmune disorders or other health conditions; i) the presence of autoimmune disorders or any other significant health conditions; ii) the existence of severe comorbidities; ii) the presence of severe comorbidities; iii) a history of immunosuppressive treatment. iii) a history of immunosuppressive treatments. The study protocols received approval from the Ethics Committee of the Second Affiliated Hospital, Zhejiang University School of Medicine, located in Hangzhou, China (approval number: 2024-1066). A total of 77 patients diagnosed with invasive lung adenocarcinoma were selected for this study. All patients had previously undergone surgical resection at this institution, and tumor samples, along with their corresponding medical data, were gathered. The Ethics Committee sanctioned this research and exempted the requirement for informed consent from the patients involved.

### Immunohistochemistry analysis

Immunohistochemistry (IHC) was performed by previously established protocols (34). The primary antibodies employed in this study included PSMD14 (dilution 1:1,000; Abcam), KI67 (dilution 1:1,000; Zhongshan Golden Bridge Biotechnology Co.), TTF1 (dilution 1:1,000; Zhongshan Golden Bridge Biotechnology Co.), CK7 (dilution 1:1,000; Zhongshan Golden Bridge Biotechnology Co.), and NapsinA (dilution 1:1,000; Zhongshan Golden Bridge Biotechnology Co.). The staining results were systematically evaluated by two pathologists, who assigned scores based on specific criteria. The tumor extent was categorized as follows: 1 (0-10%), 2 (11-20%), 3 (21-30%), 4 (31-40%), 5 (41-50%), 6 (51-60%), 7 (61-70%), 8 (71-80%), 9 (81-90%), and 10 (91-100%).

### Statistical analysis

Statistical and bioinformatics evaluations were performed utilizing R software (version 4.2.0). A one-way analysis of variance (ANOVA) was employed to assess differences among multiple groups, which was subsequently followed by Tukey’s *post hoc* test for pairwise analysis. In cases where comparisons were made between two groups, the Wilcoxon rank-sum test was applied. Additionally, correlation analyses were conducted using both Spearman’s and distance correlation techniques. For the analysis of survival data, the KM method was utilized. The survival duration was defined as the interval from the initiation of treatment to the conclusion of the observation period.

## Results

### Expression of JAMM family genes in LUAD

**Figure 1:**
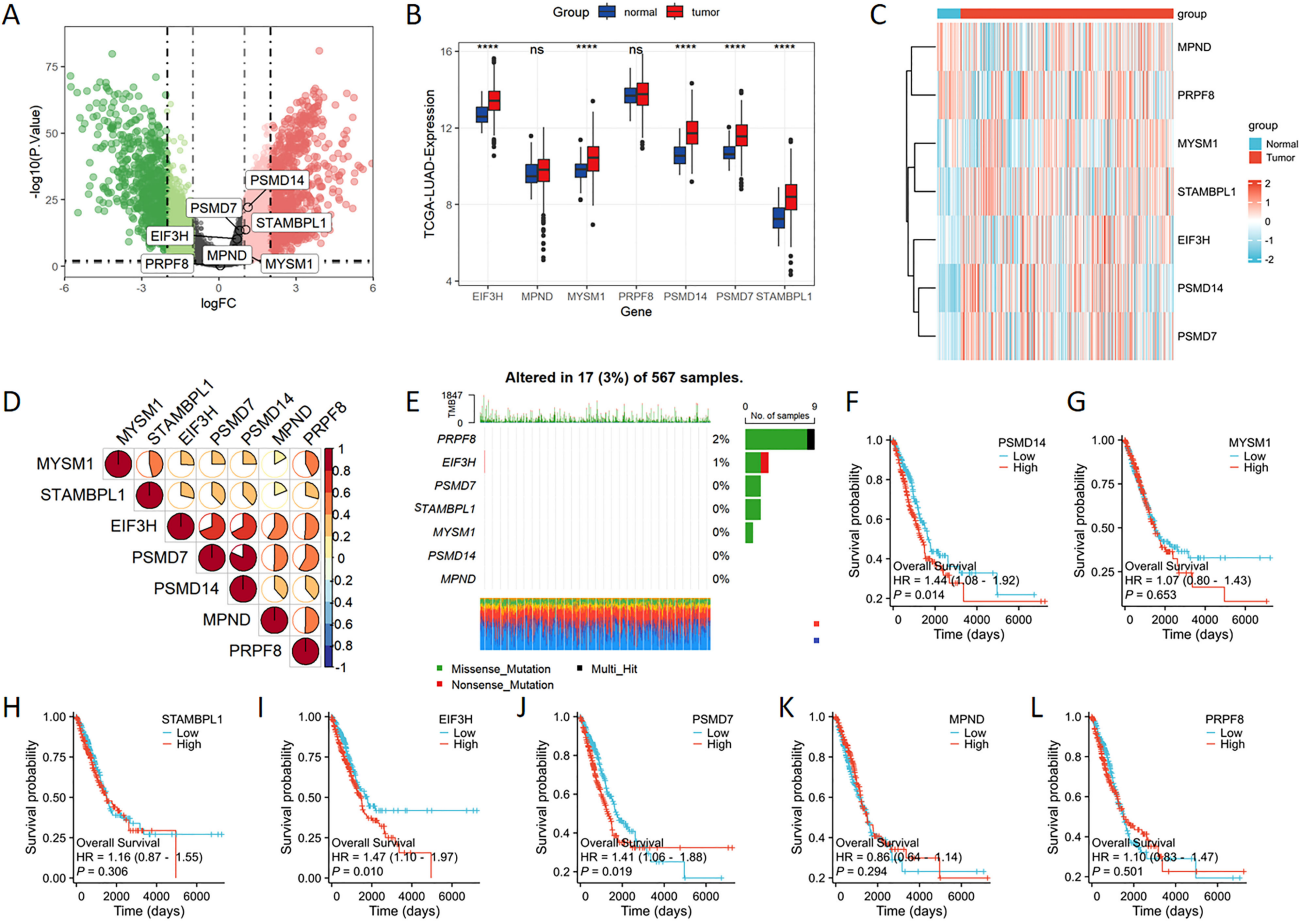
The expression levels of JAMM family genes in LUAD **(A)** The volcano plot highlights genes that exhibit significant upregulation (represented in red) and downregulation (depicted in green). **(B)** The expression levels of EIF3H, MYSM1, PSMD14, PSMD7, and STAMBPL1 were markedly increased in the tumor cohort. **(C)** The heatmap provides a visual representation of the expression patterns of JAMM family genes. **(D)** Spearman’s correlation analysis reveals the interrelationships among the JAMM family genes. **(E)** The mutational landscape of the JAMM family genes in LUAD is presented. **(F-L)** Kaplan-Meier survival analysis evaluates the association between JAMM family gene expression and patient prognosis. ****, *p* < 0.0001; ns, not significant.

The volcano plot effectively depicts the differentially expressed genes belonging to the JAMM family in LUAD. The findings suggest that PSMD14 and STAMBPL1 are among the genes showing differential expression in LUAD, with both exhibiting heightened expression levels (**Figure 1A**). Furthermore, the expression levels of EIF3H, MYSM1, PSMD14, PSMD7, and STAMBPL1 in LUAD tissues are significantly higher than those observed in normal tissues (**Figure 1B**). These observations are corroborated by the gene heatmap provided (**Figure 1C**). Through Spearman’s correlation analysis, we identified positive correlations among the JAMM family genes (**Figure 1D**). In addition, we investigated the mutational landscape of the JAMM family genes in LUAD, which revealed a low mutation frequency (**Figure 1E**). Finally, elevated expression levels of PSMD14, EIF3H, and PSMD7 were found to be significantly linked to poorer clinical outcomes (**Figures 1F–L**).

### Construction of prognostic risk model

**Figure 2:**
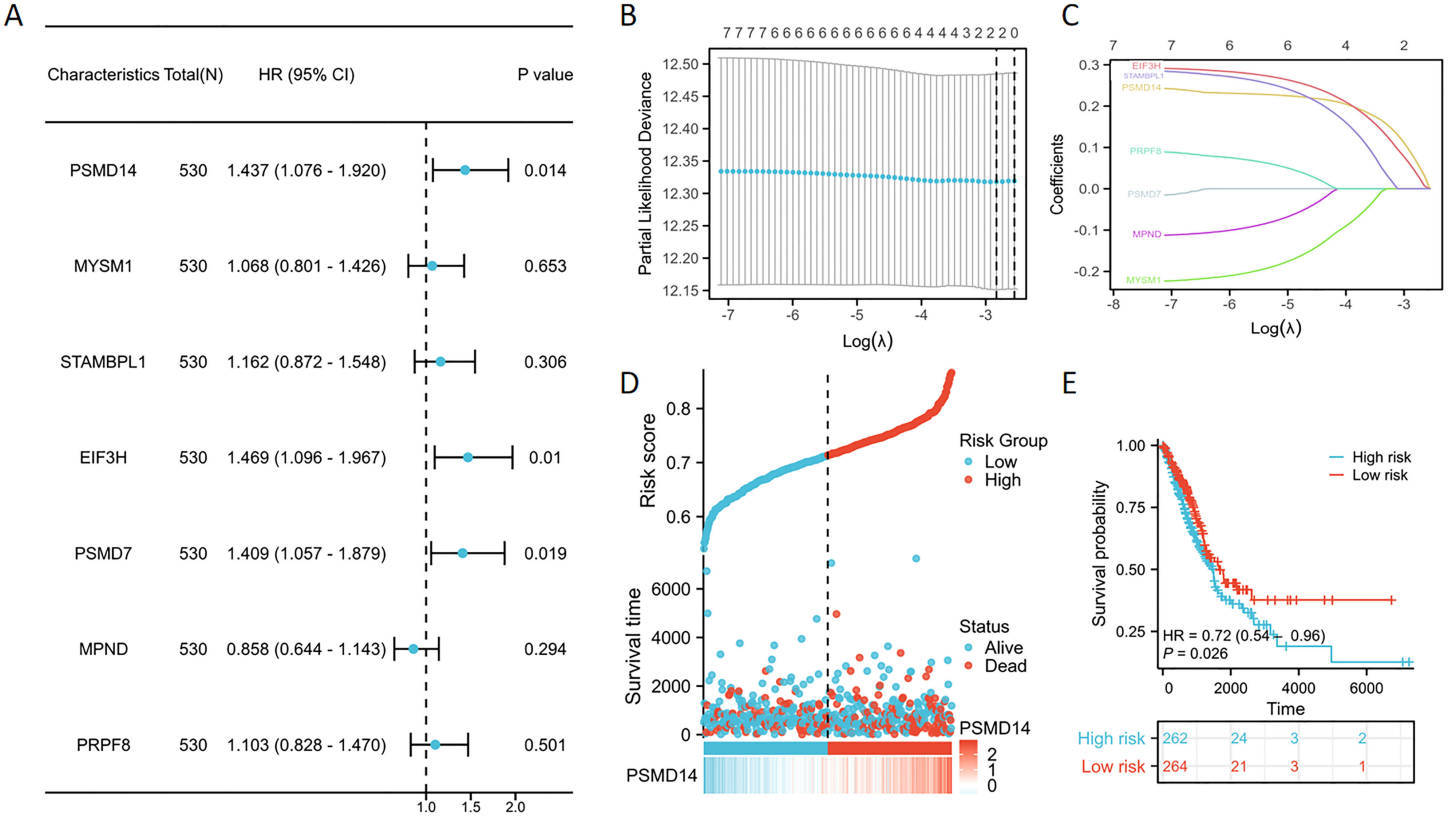
Identification of prognostic genes in LUAD linked to JAMM utilizing lasso Cox regression **(A)** Forest plot demonstrates the prognostic relevance of genes belonging to the JAMM family. **(B, C)** LASSO regression analysis was performed on genes correlated with JAMM. **(D)** The distribution of expression profiles for the seven JAMM family genes, as well as their associated risk scores and survival statuses, is presented. **(E)** A survival analysis comparing high-risk and low-risk groups is illustrated.

Univariate Cox regression analysis was utilized to evaluate the prognostic significance of genes belonging to the JAMM family (**Figure 2A**). The findings revealed that individuals with low expression levels of PSMD14, EIF3H, and PSMD7 experienced a notable survival advantage compared to those exhibiting high expression levels of these genes. Subsequently, through LASSO Cox regression analysis, we identified three pivotal genes that displayed the most favorable prognostic value—PSMD14, EIF3H, and PSMD7 (**Figures 2B, C**). Additionally, we analyzed the expression patterns of seven JAMM family genes, alongside risk scores, survival duration, and survival status within the TCGA dataset. Our analysis underscored the considerable prognostic relevance of PSMD14 (**Figure 2D**). Moreover, Kaplan-Meier survival analysis indicated that the high-risk cohort demonstrated significantly worse prognostic outcomes in comparison to the low-risk cohort (**Figure 2E**).

### PSMD14 expression in various tumor tissues

**Figure 3:**
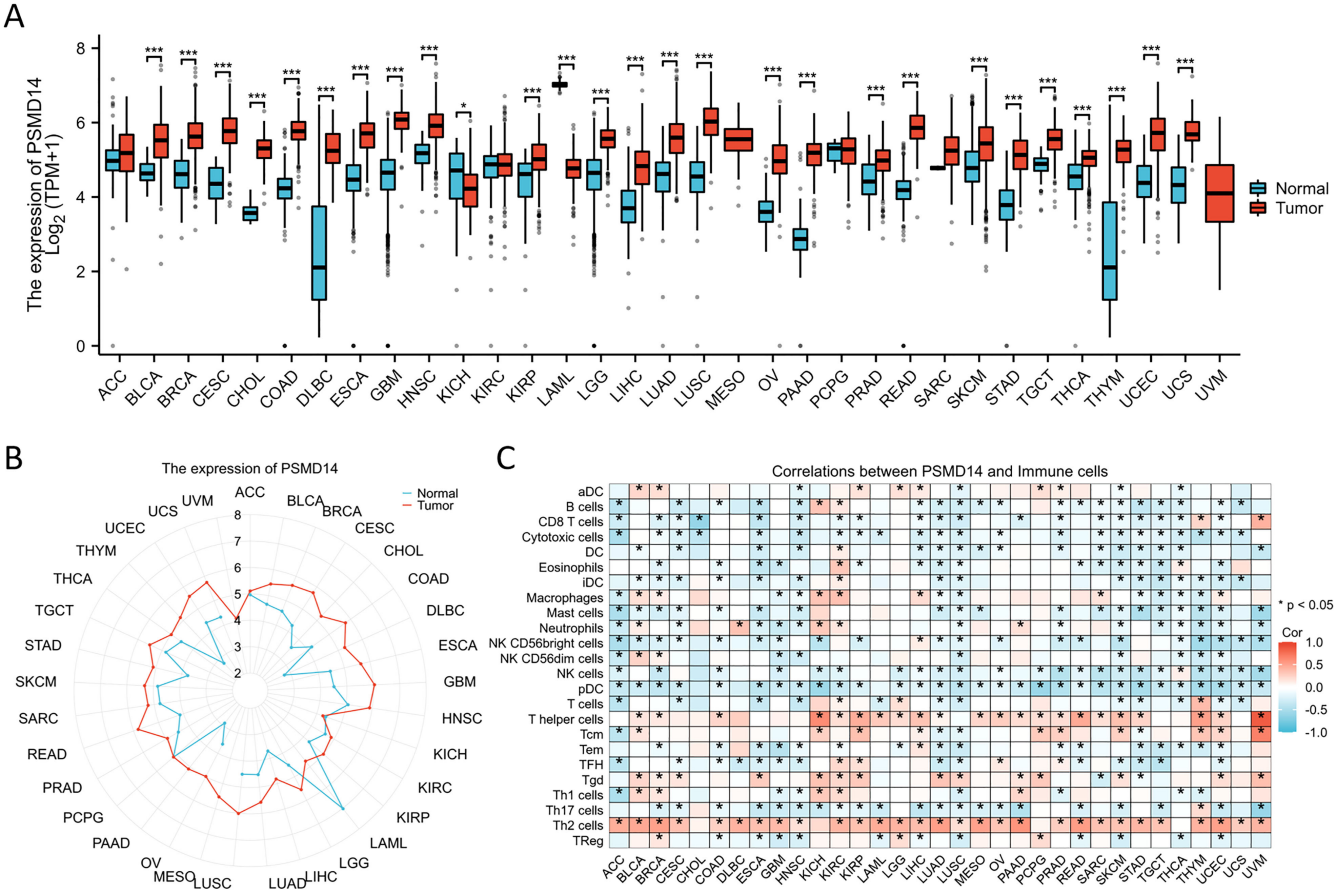
The expression levels of PSMD14 across different tumor types **(A, B)** The expression levels of PSMD14 in tumor tissues compared to normal tissues were analyzed across various cancer types using data obtained from TCGA and the GTEx databases. **(C)** The association between the expression of PSMD14 and the extent of immune cell infiltration in various malignancies. *, *p*<0.05; ***, *p*<0.001.

Given the limited availability of corresponding normal tissue expression data within the TCGA database, we subsequently performed a comprehensive analysis utilizing matched normal tissue expression data obtained from the GTEx database to enhance the reliability of our findings. Our analysis revealed that PSMD14 expression levels were notably increased across a majority of cancer types, including bladder cancer (BLCA), breast cancer (BRCA), cervical squamous cell carcinoma (CESC), cholangiocarcinoma (CHOL), colon adenocarcinoma (COAD), diffuse large B-cell lymphoma (DLBC), esophageal carcinoma (ESCA), glioblastoma multiforme (GBM), head and neck squamous cell carcinoma (HNSC), kidney renal papillary cell carcinoma (KIRP), lower grade glioma (LGG), liver hepatocellular carcinoma (LIHC), LUAD, lung squamous cell carcinoma (LUSC), ovarian cancer (OV), pancreatic adenocarcinoma (PAAD), prostate adenocarcinoma (PRAD), rectum adenocarcinoma (READ), skin cutaneous melanoma (SKCM), stomach adenocarcinoma (STAD), testicular germ cell tumors (TGCT), thyroid carcinoma (THCA), thymoma (THYM), uterine corpus endometrial carcinoma (UCEC), and uterine carcinosarcoma (UCS). Conversely, a significant reduction in PSMD14 expression was noted in the tumor tissues of the kidney chromophobe (KICH) (**Figures 3A, B**). Furthermore, our findings indicated a strong association between PSMD14 expression and immune infiltration, with a notable correlation observed particularly with T helper cells and Th2 cells (**Figure 3C**). Collectively, these results imply that PSMD14 may play a critical role within the tumor microenvironment and could be implicated in the initiation and progression of tumors.

### PSMD14 expression level correlates with prognosis in cancers

**Figure 4:**
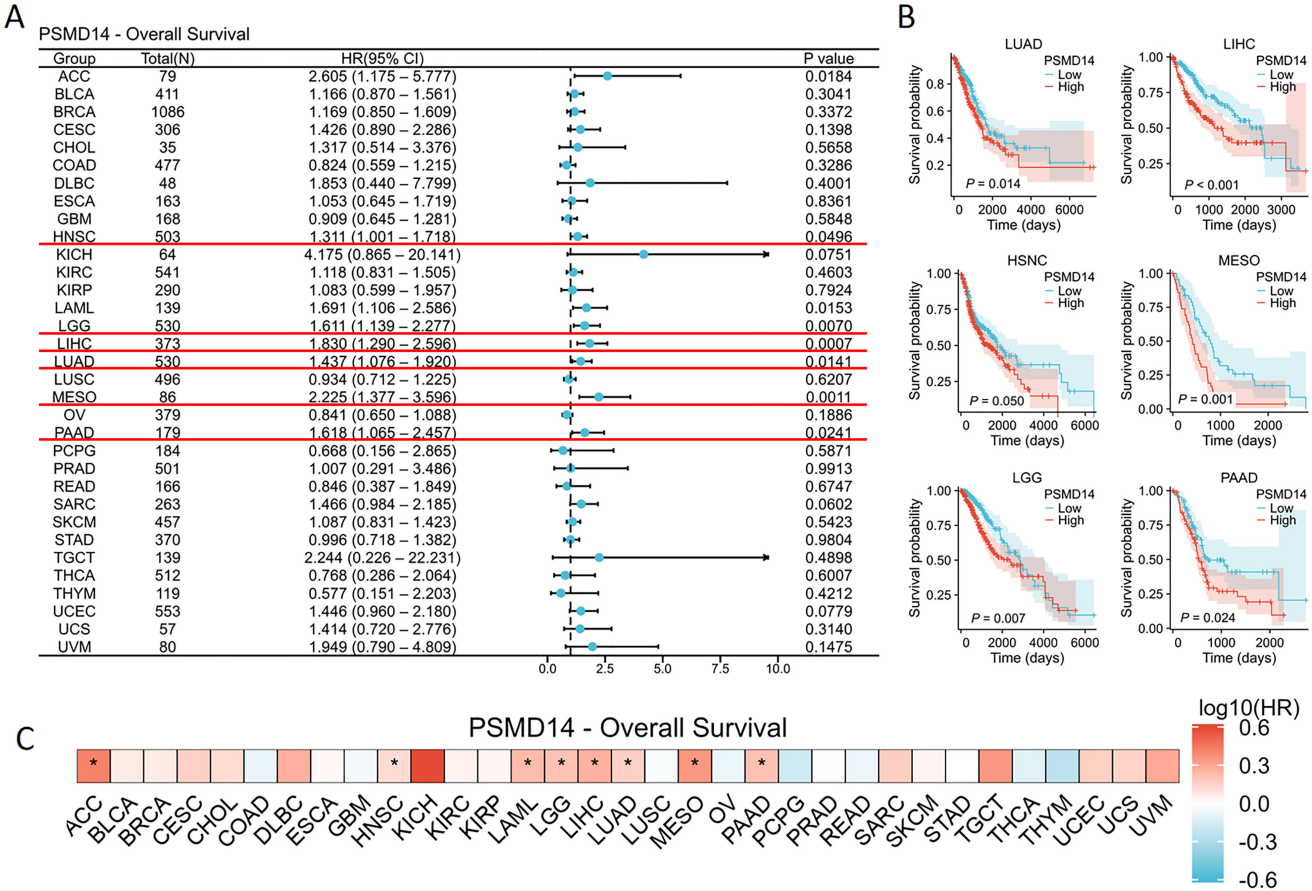
Survival analysis of the PSMD14 gene across various cancer types utilizing the TCGA database **(A)** The relationship between the expression levels of PSMD14 and OS in patients across various cancer types was assessed through the univariate Cox regression analysis. **(B)** Kaplan-Meier survival curves demonstrating OS with statistically significant results across six distinct cancer types within the TCGA dataset. **(C)** The relationship between the expression levels of PSMD14 and OS in patients diagnosed with various types of cancer was assessed through a heatmap analysis. *, *p*<0.05.

To gain deeper insights into the impact of PSMD14 expression on cancer patient prognosis, we acquired RNA sequencing and clinical data from TCGA. A univariate Cox regression analysis was conducted to assess the correlation between PSMD14 expression levels and OS across 33 distinct cancer types, as illustrated in **Figure 4A**. Notably, elevated PSMD14 expression was markedly strongly linked to unfavorable poor prognoses in patients diagnosed with HNSC, LGG, LIHC, LUAD, mesothelioma (MESO), and PAAD, with the most pronounced association observed in PAAD (**Figure 4B**). Furthermore, these results are reinforced are corroborated by the heatmap representation (**Figure 4C**).

### Clinical prognostic value of PSMD14 in lung cancer

**Figure 5:**
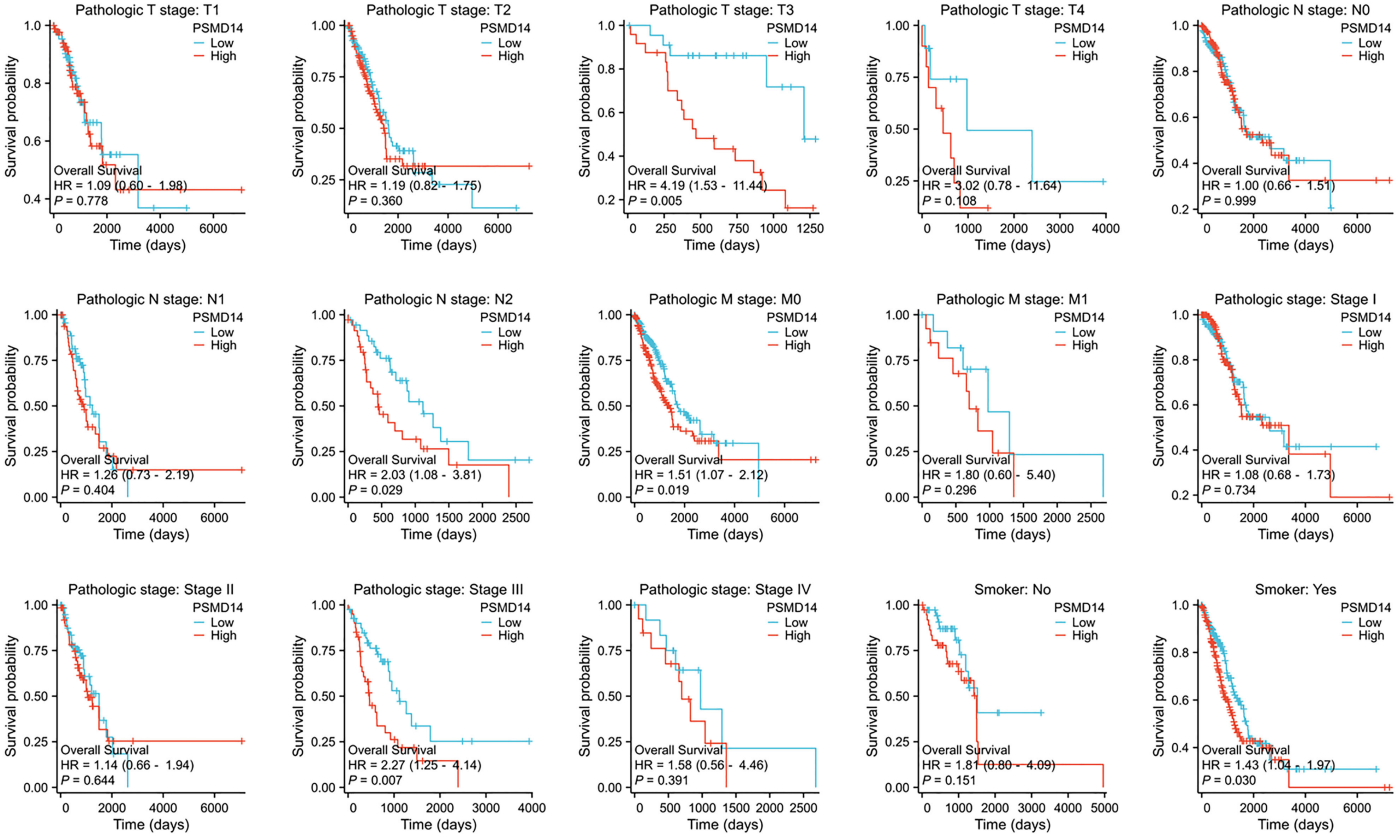
The association between OS and the expression levels of PSMD14 in various clinical subgroups of LUAD.

In the domain of lung cancer research, the expression level of PSMD14 has been identified as having a strong relationship with patient prognosis. Consequently, this investigation aims to further examine the association between PSMD14 expression and prognosis within various clinical subgroups. The findings indicate that elevated PSMD14 expression is markedly linked to reduced OS across different clinical subpopulations, especially among individuals classified as T3 stage, N2 stage, M0 stage, those with pathological stage III, and among smokers (**Figure 5**).

### GO and KEGG analysis of PSMD14

**Figure 6:**
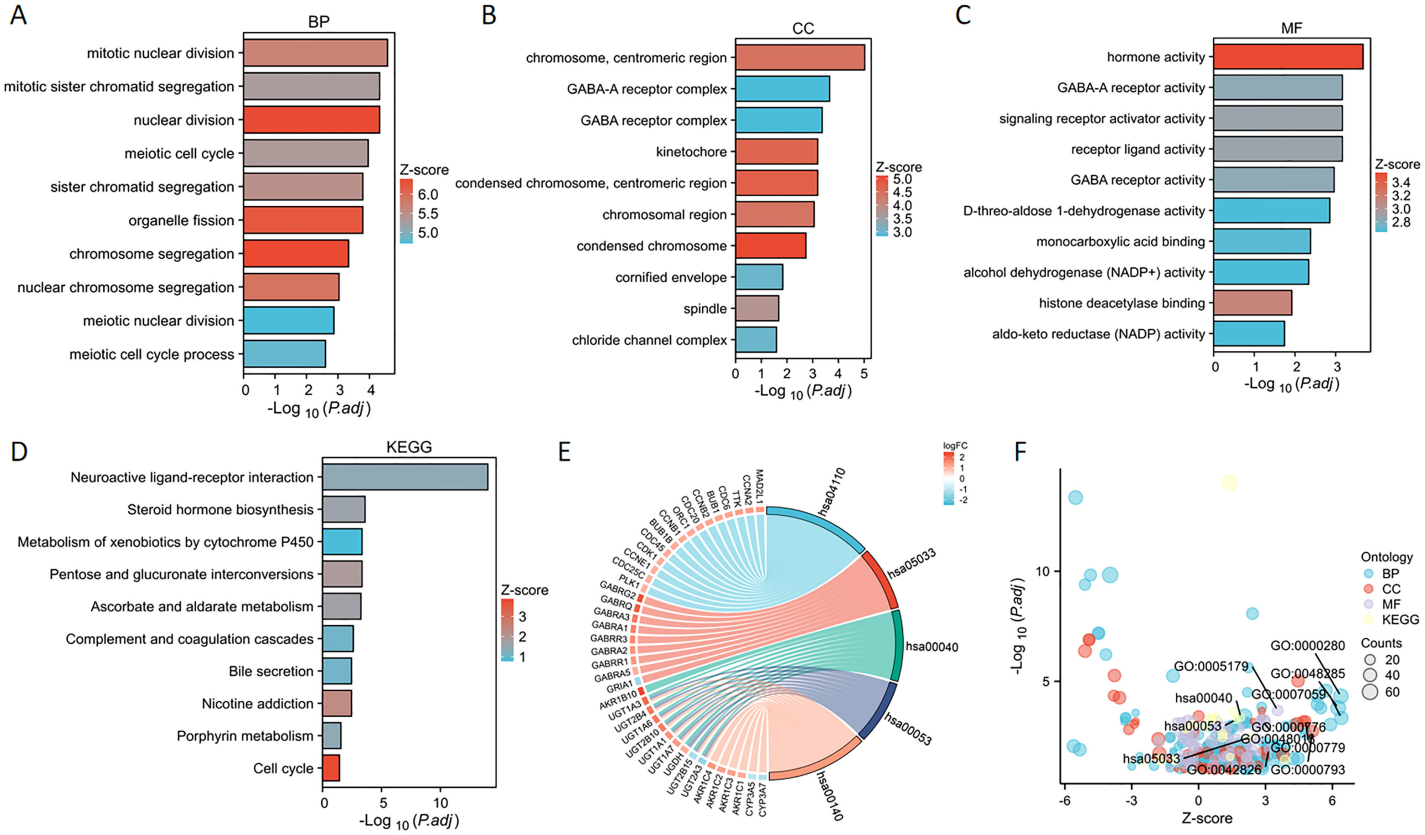
GO and KEGG enrichment analysis of PSMD14 **(A–C)** Enrichment analyses of Gene Ontology terms associated with PSMD14 in LUAD have been conducted. **(D)** The enrichment analysis of KEGG pathways associated with PSMD14 in LUAD was performed. **(E)** The representation of outcomes derived from the KEGG chord diagram analysis. **(F)** The bubble plot illustrates the scores of enrichment.

We performed enrichment analyses for GO and KEGG pathways to elucidate the biological roles of PSMD14. The GO analysis indicated notable enrichment in various biological processes, including chromosome segregation, nuclear division, organelle fission, and sister chromatid segregation (**Figures 6A, F**). The analysis highlighted essential cellular components such as condensed chromosomes, centromeric regions, kinetochores, spindles, cornified envelopes, chloride channel complexes, and GABA receptor complexes (**Figures 6B, F**). Additionally, significant enrichment was observed in molecular functions related to hormone activity, histone deacetylase binding, signaling receptor activator activity, GABA receptor activity, monocarboxylic acid binding, D-threo-aldose 1-dehydrogenase activity, alcohol dehydrogenase (NADP+) activity, and aldo-keto reductase (NADP) activity (**Figures 6C, F**). The KEGG pathway enrichment analysis revealed substantial enrichment in pathways such as the cell cycle, nicotine addiction, pentose and glucuronate interconversions, ascorbate, and alternate metabolism, steroid hormone biosynthesis, porphyrin metabolism, neuroactive ligand-receptor interactions, complement and coagulation cascades, bile secretion, and the metabolism of xenobiotics by cytochrome P450 (**Figures 6D–F**) (**Table 1**). In conclusion, the involvement of PSMD14 in the progression of lung cancer necessitates further investigation.

**Table 1:** Supplementary information of GO and KEGG analysis.

Ontology	ID	Description	pvalue
BP	GO:0007059	chromosome segregation	2.26845e-06
BP	GO:0000280	nuclear division	1.42133e-07
BP	GO:0048285	organelle fission	6.14623e-07
CC	GO:0000793	condensed chromosome	6.9472e-05
CC	GO:0000779	condensed chromosome, centromeric region	1.42473e-05
CC	GO:0000776	kinetochore	1.53214e-05
MF	GO:0005179	hormone activity	2.68129e-07
MF	GO:0042826	histone deacetylase binding	0.000683647
MF	GO:0048018	receptor ligand activity	3.4414e-06
KEGG	hsa04110	Cell cycle	0.001879616
KEGG	hsa05033	Nicotine addiction	0.000146753
KEGG	hsa00040	Pentose and glucuronate interconversions	6.43111e-06
KEGG	hsa00053	Ascorbate and aldarate metabolism	1.21256e-05
KEGG	hsa00140	Steroid hormone biosynthesis	1.68807e-06

### GSEA analysis of PSMD14

**Figure 7:**
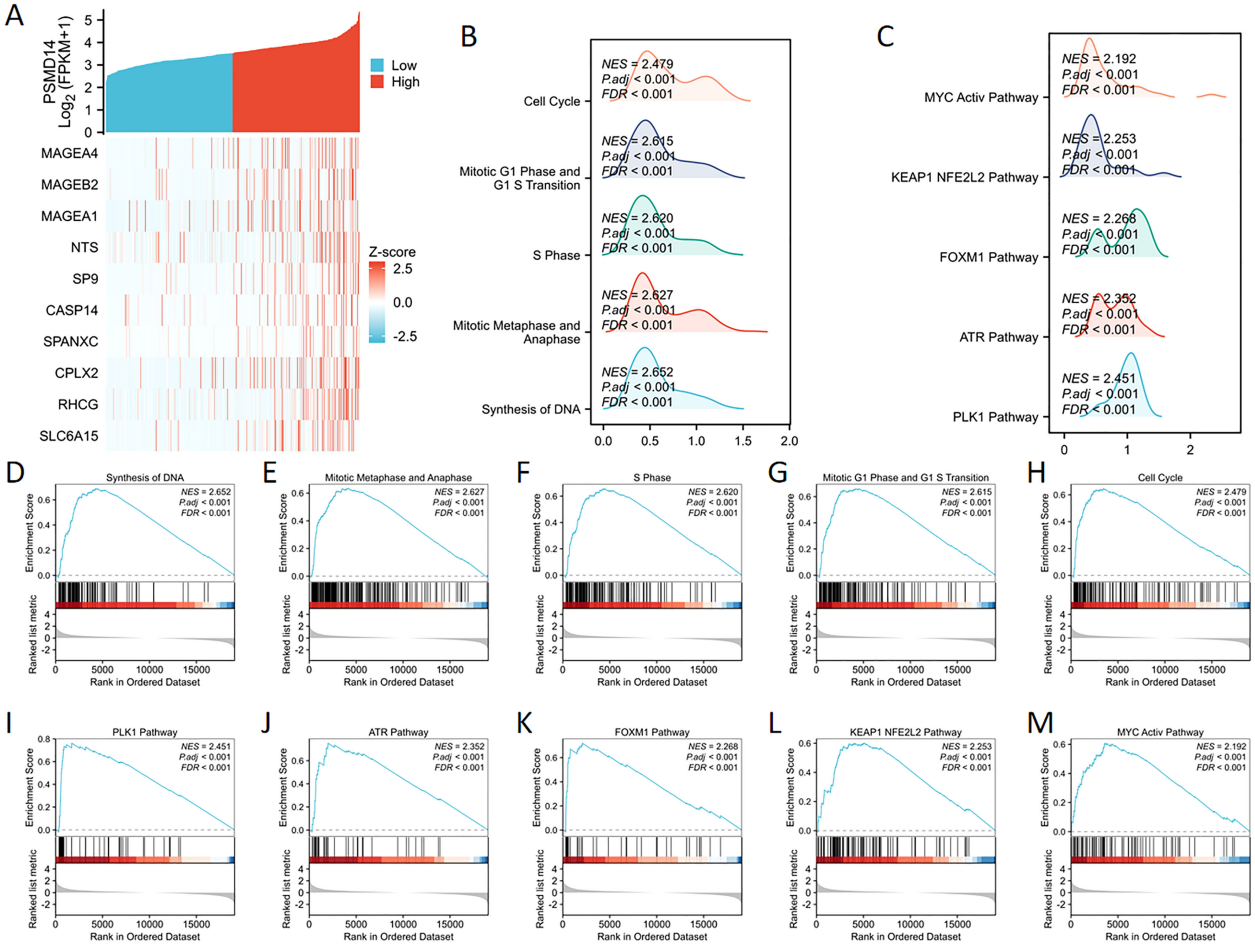
GSEA enrichment analysis of PSMD14 **(A)** The expression levels of the PSMD14 gene were employed to generate a heat map illustrating ten genes that showed increased expression. **(B, C)** Mountain plots visually represent the results of GSEA enrichment. **(D–M)** Investigation into the functional contributions and pathway enrichment linked to the PSMD14.

We undertook a comprehensive analysis of the expression profile of the PSMD14 gene to deepen our understanding of its biological relevance in LUAD. The generated gene expression heat map illustrated the ten genes that displayed significant deviations in expression levels (with a log fold change greater than |1| and a p-value of less than 0.05) (**Figure 7A**). To assess the influence of varying levels of PSMD14 expression on the progression of lung cancer, we employed GSEA to pinpoint the biological functions and pathways that were most significantly enriched. The findings revealed a marked enrichment of the high PSMD14 expression group in several biological processes, including DNA synthesis, mitotic metaphase, anaphase, S phase, mitotic G1 phase, G1/S transition, and overall cell cycle progression (**Figures 7B, D–H**). Furthermore, the high PSMD14 expression group demonstrated substantial enrichment in notable pathways such as those involving PLK1, ATR, FOXM1, KEAP1-NFE2L2, and MYC activation (**Figures 7C, I–M**).

### The association between PSMD14 gene expression and immune cell infiltration

**Figure 8:**
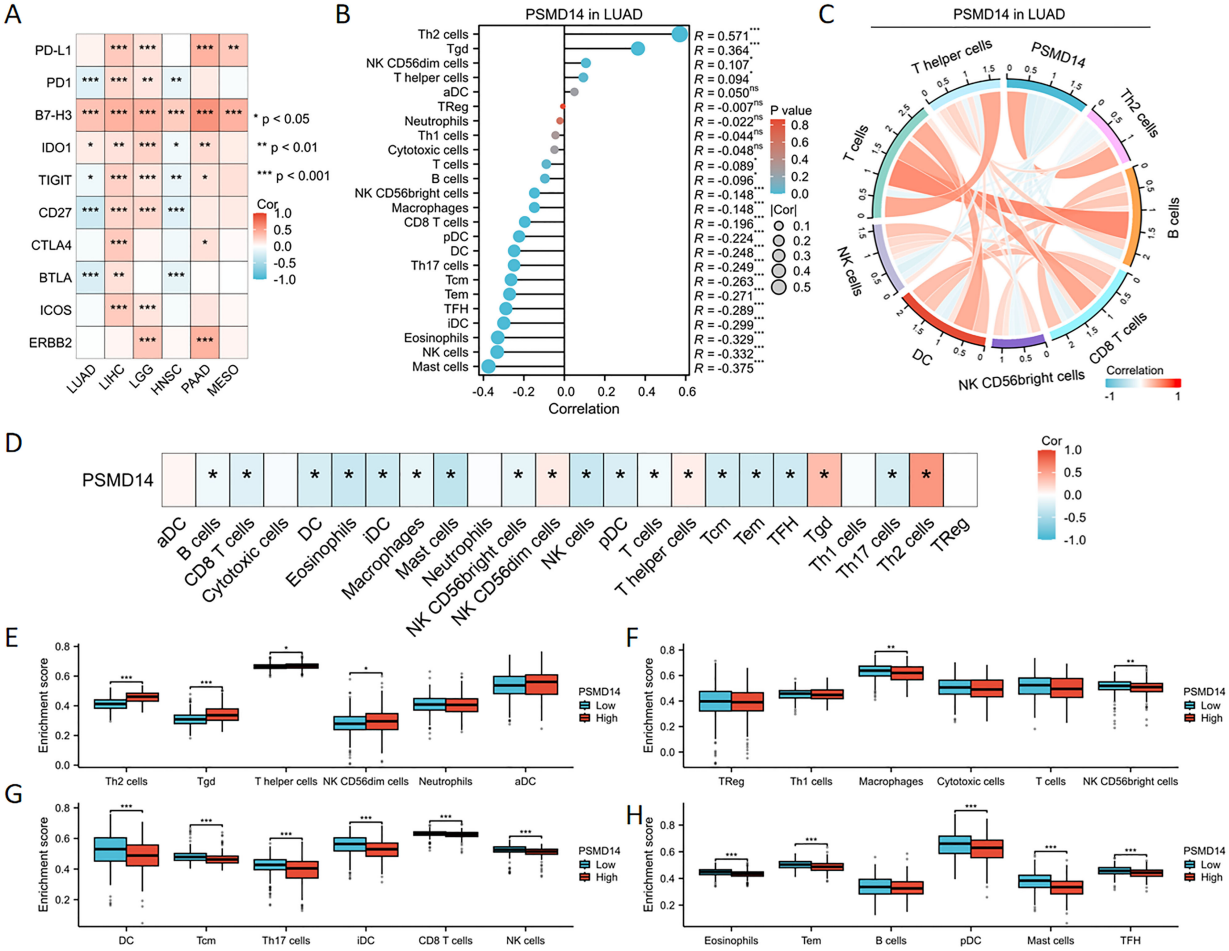
Association between PSMD14 gene expression and infiltration of immune cells **(A)** The expression of PSMD14 exhibited a significant association with immune checkpoint levels across a range of tumor types. **(B)** The relationship between the expression levels of the PSMD14 gene and the infiltration status of immune cells. **(C)** The chord diagram visually represents the relationship between PSMD14 and various immune cell types. **(D)** The heatmap demonstrates the relationship between PSMD14 and various immune cell types. **(E-H)** The degree of enrichment of particular immune cell subsets varied between the groups with high and low expression levels of the PSMD14 gene. *, *p*<0.05; **, *p*<0.01; ***, *p*<0.001.

The expression of PSMD14 exhibited a negative correlation with the levels of immune checkpoint molecules in LUAD, particularly with PD-1, TIGIT, CD27, and BTLA, as illustrated in **Figure 8A**. To further elucidate the characteristics of the immune microenvironment in lung cancer, we analyzed immune cell infiltration within the tumor microenvironment. Utilizing the single-sample Gene Set Enrichment Analysis (ssGSEA) algorithm, we evaluated the Pearson correlation coefficients between traits of the immune microenvironment and PSMD14 expression. Our findings indicate a positive relationship between PSMD14 expression and the infiltration of Th2 cells, Tgd cells, and various other immune cell types, as represented in **Figures 8B, D**. In contrast, a negative correlation was observed between PSMD14 expression and the infiltration levels of eosinophils, NK cells, mast cells, and other cellular constituents. Additionally, chord plot analysis revealed a pronounced correlation between PSMD14 expression and the presence of T cells, B cells, NK cells, and dendritic cells (DCs), as depicted in **Figure 8C**. The relationship between the immune microenvironment and enrichment scores was further assessed by constructing immune cell profiles derived from LUAD samples. Our results demonstrate a notable reduction in the enrichment scores of the majority of immune cells within the high PSMD14 expression cohort, as shown in **Figures 8E, H**.

### Association of PSMD14 expression with clinical parameters in TCGA database

**Figure 9:**
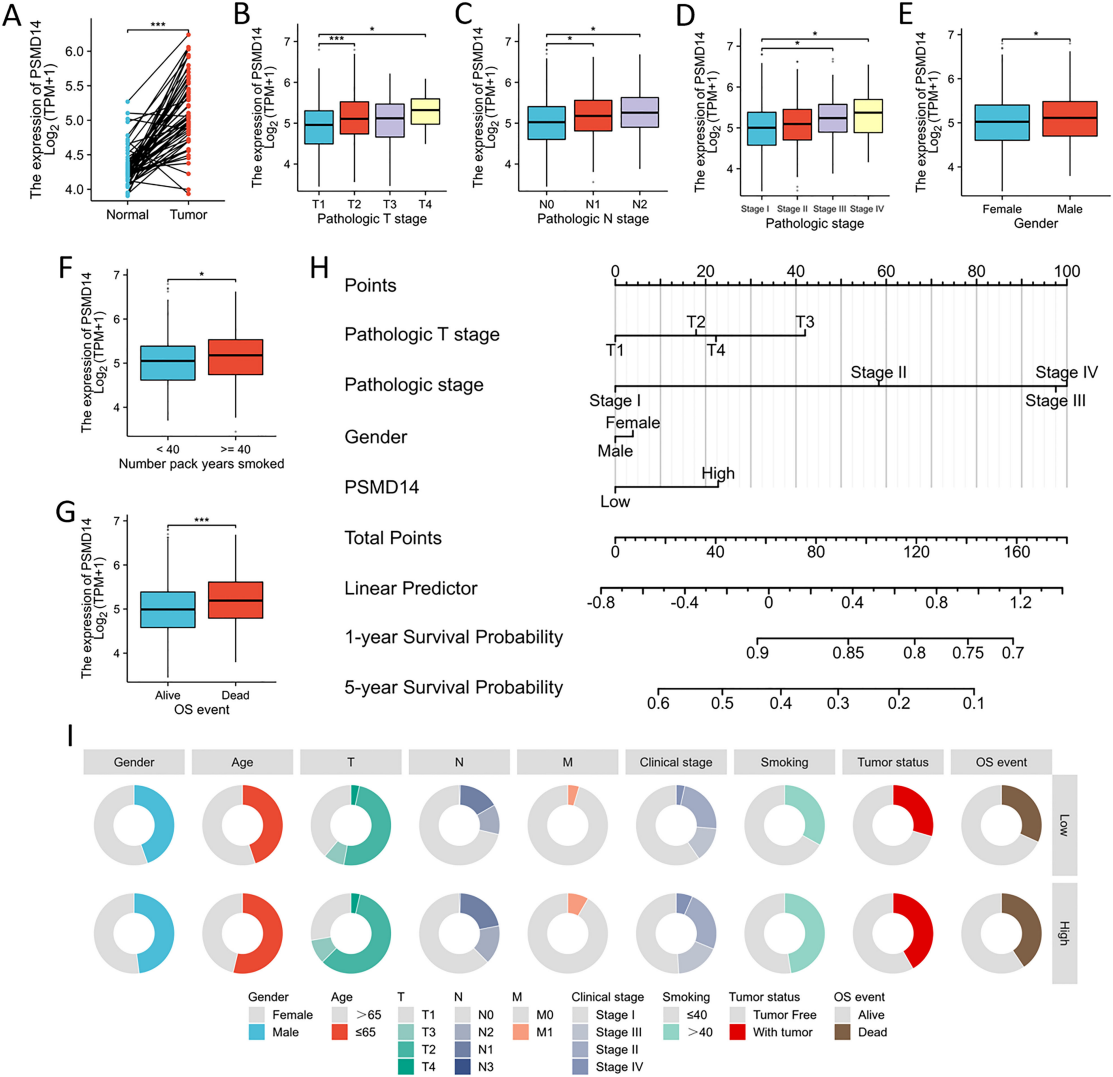
Association between PSMD14 expression and the clinical-pathological parameters **(A)** The expression level of PSMD14 mRNA was markedly increased in LUAD tissues. **(B–G)** The relationship between the expression of PSMD14 and various clinical parameters, including T stage, N stage, pathological stage, gender, cumulative smoking history measured in pack-years, and OS events, has been investigated. **(H)** Nomogram models for survival prediction have been developed to estimate the OS rates of patients diagnosed with LUAD over a timeframe of 1 and 5 years. **(I)** The diagram illustrates the correlation between the expression levels of PSMD14 and various clinical-pathological characteristics associated with LUAD. *, *p*<0.05; ***, *p*<0.001.

**Figure 10:**
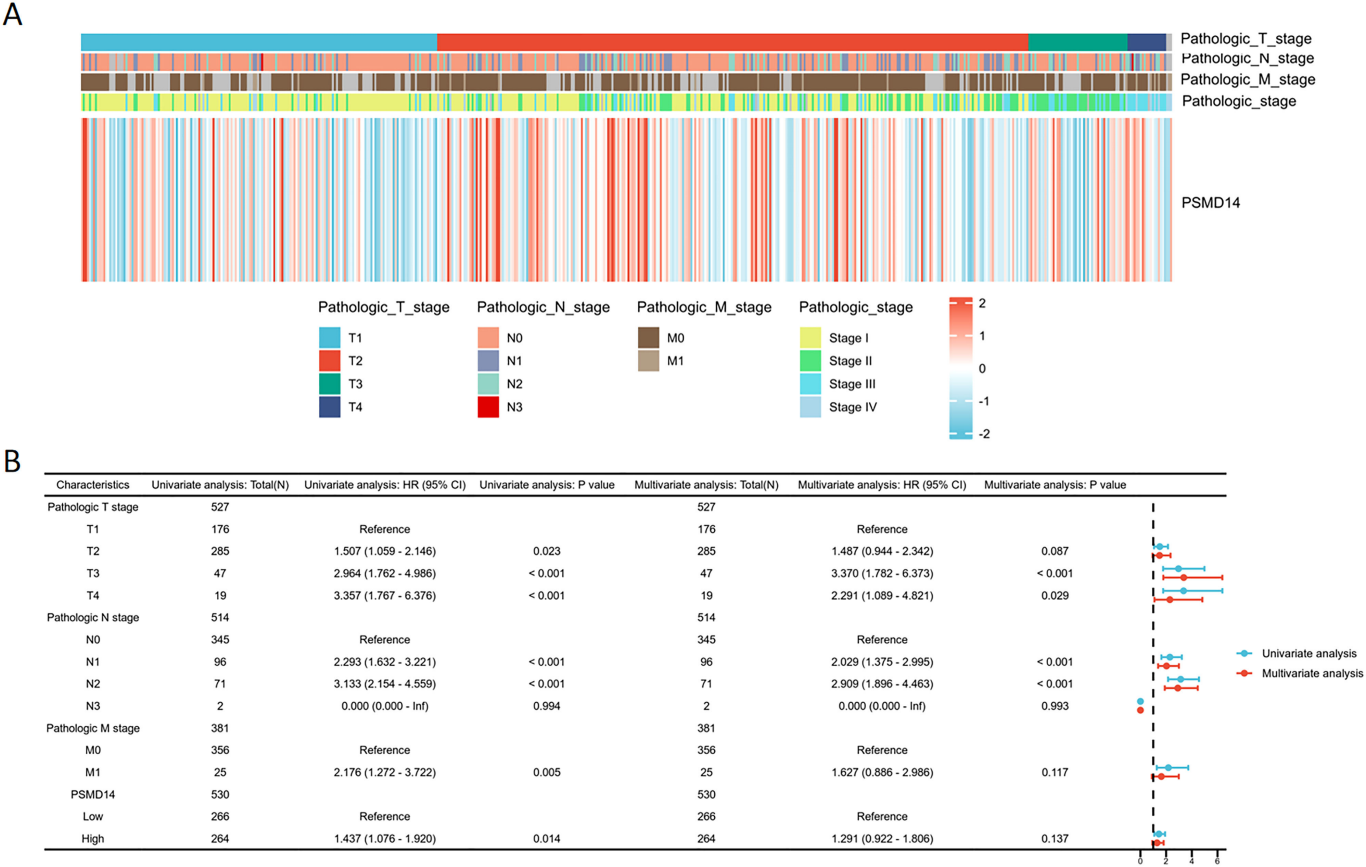
Logistic regression analysis of PSMD14 expression **(A)** The heatmap demonstrates the relationship between PSMD14 and various clinical parameters. **(B)** Univariate and multivariate Cox regression analyses were employed to examine the relationships between OS and various clinical variables.

In the TCGA dataset, the mRNA levels of PSMD14 were markedly increased in tumor tissues (**Figure 9A**). A positive correlation was observed between elevated PSMD14 expression and factors such as higher T stage, N stage, pathological stage, and the total number of pack years smoked (**Figures 9B–G, I**). Additionally, heightened PSMD14 expression was linked to diminished OS outcomes (illustrated in **Figures 9F, I**). We formulated a clinical prognostic risk score specifically for LUAD, which encompassed the T stage, pathological stage, gender, and PSMD14 expression levels (**Figure 9H**). The expression of PSMD14 holds promise for improving the precision of survival forecasts at both 1- and 5-year intervals. Correlations between the clinicopathological characteristics of LUAD patients and their PSMD14 protein levels are presented in **Table 2**. Collectively, PSMD14 expression exhibited a significant relationship with the prognosis of LUAD patients. The gene expression heatmap, which integrates various clinical parameters, demonstrates that heightened PSMD14 expression is substantially associated with advanced T, N, M, and pathological stages (**Figure 10A**). Univariate Cox regression analysis identified T stage, N stage, M stage, and PSMD14 expression as independent prognostic indicators for LUAD patients. Moreover, multivariate Cox regression analysis confirmed that T and N stages serve as independent prognostic factors (**Figure 10B**).

**Table 2:** Association of PSMD14 expression with clinicopathological characteristics in patients with LUAD in TCGA database.

Characteristics	Low expression of PSMD14	High expression of PSMD14	pvalue	Method
n	66	68		
T stage, n (%)			0.048	Yates' correction
T1	20 (14.9%)	9 (6.7%)		
T2	39 (29.1%)	43 (32.1%)		
T3	5 (3.7%)	11 (8.2%)		
T4	2 (1.5%)	5 (3.7%)		
N stage, n (%)			0.378	Chisq test
N0	45 (33.6%)	39 (29.1%)		
N1	13 (9.7%)	20 (14.9%)		
N2	8 (6%)	9 (6.7%)		
M stage, n (%)			0.248	Chisq test
M0	62 (46.3%)	60 (44.8%)		
M1	4 (3%)	8 (6%)		
Histologic grade, n (%)			0.047	Chisq test
I	34 (25.4%)	23 (17.2%)		
II	22 (16.4%)	24 (17.9%)		
III	10 (7.5%)	21 (15.7%)		
event, n (%)			0.033	Chisq test
Alive	44 (32.8%)	33 (24.6%)		
Dead	22 (16.4%)	35 (26.1%)		

### Single−cell RNA−seq profiling and clustering

**Figure 11:**
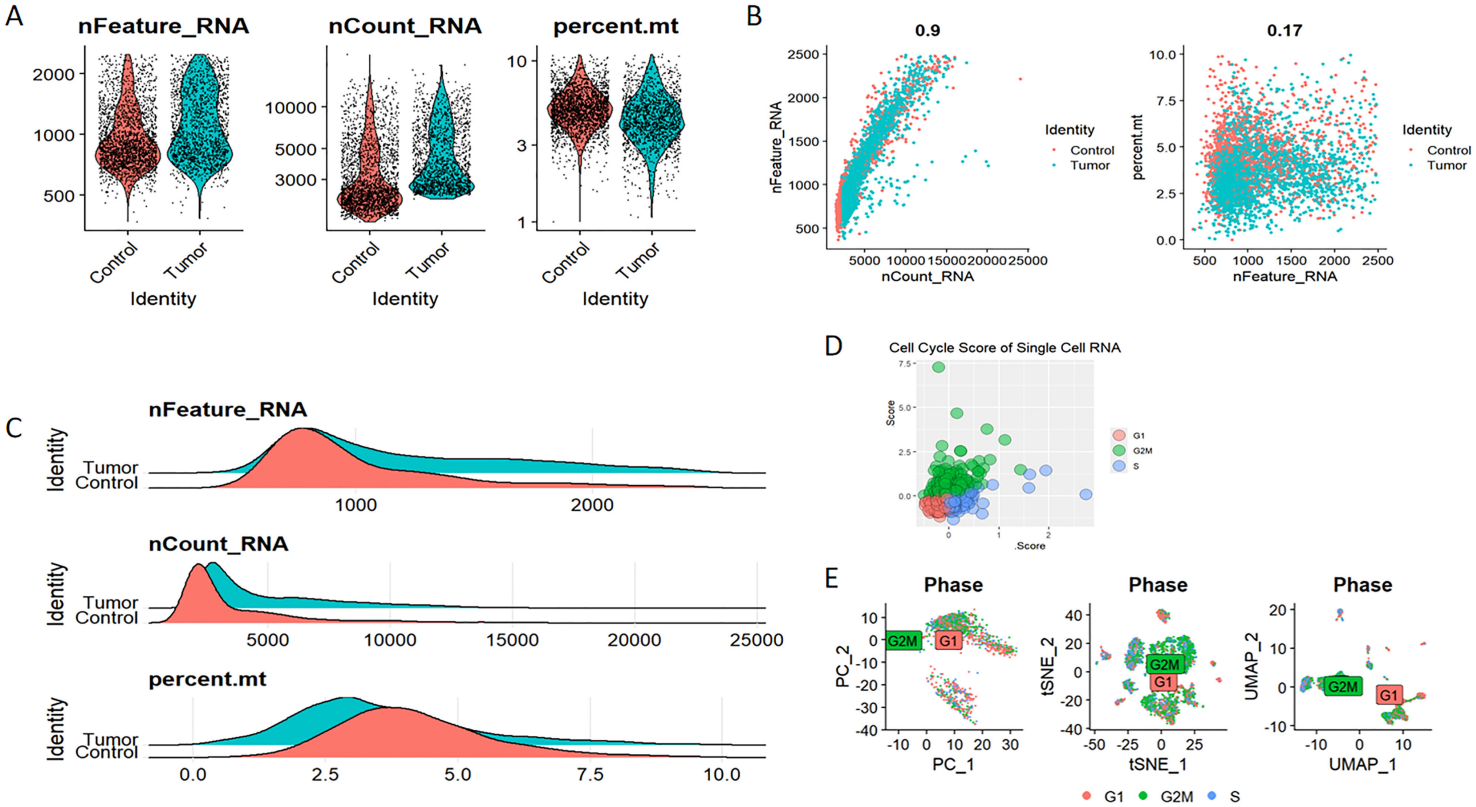
Eliminating the impact of mitochondrial DNA and cell cycle on single-cell data analysis **(A–C)** The assessment of RNA characteristics encompasses nFeature_RNA, nCount_RNA, and the proportion of mitochondrial content (percent.mt). **(D)** Cell cycle score of single-cell RNA. **(E)** Analysis of cell clustering across various stages of the cell cycle.

**Figure 12:**
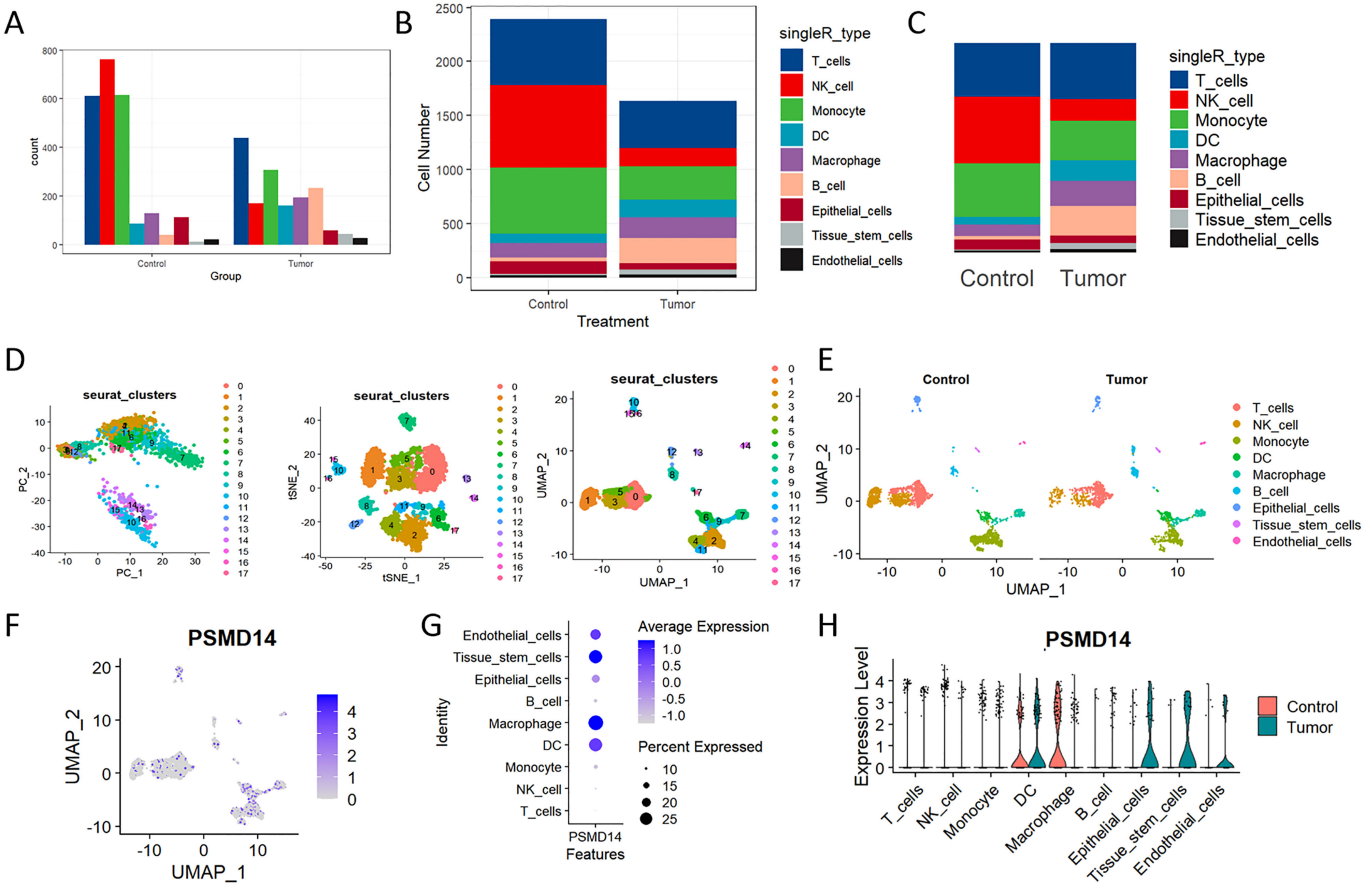
Analysis of single-cell RNA sequencing of NSCLC tissues **(A, B)** The bar chart, along with the stacked bar chart, illustrates the cell counts for both the control group and the tumor group. **(C)** The distribution of each cell type within the control and tumor cohorts. **(D)** PC, tNSE, and UMAP of the 18 distinct cellular clusters. **(E)** The clusters underwent additional annotation utilizing specific marker genes, which encompassed T cells, NK cells, monocytes, macrophages, DC, B cells, tissue stem cells, as well as endothelial and epithelial cells. **(F)** The UMAP visualization illustrates the expression levels of PSMD14 distributed among various cell clusters. **(G)** The expression of PSMD14 across all recognized cell types. **(H)** Violin plots illustrating the expression levels of PSMD14 across all recognized cell types.

Elevated expression levels of mitochondrial genes may indicate potential cell damage or trigger apoptosis. Consequently, it is critical to mitigate the effects of mitochondrial gene expression during the analysis of single-cell data. For clustering analysis, we established the following criteria: nFeature_RNA > 200, nFeature_RNA < 2500, and percent.mt < 10, nFeature_RNA > 200, nFeature_RNA < 2500, and percent.mt < 10, aimed at reducing their possible interference with subsequent analyses (**Figures 11A–C**). The Seurat package compiles marker genes corresponding to the G1, G2M, and S phases of the cell cycle and allocates scores to individual cells, thereby facilitating the assessment of their phase-specific scores (**Figure 11D**). In comparison to PCA and t-SNE for dimensionality reduction, UMAP demonstrates superior clustering performance owing to its_direct nonlinear dimensionality reduction methodology (**Figure 11E**). Within the GSE117570 dataset, which encompasses four paired NSCLC tissue samples, we observed a significant reduction in the populations of T cells, NK cells, and monocytes within the tumor group, contrasted by a notable increase in B cells (**Figures 12A–C**). The scRNA-seq data were visualized through PC, tSNE, and UMAP techniques, successfully categorizing the cells into 18 distinct clusters (**Figure 12D**). Each cluster was annotated utilizing the SingleR R software package to identify cell types (**Figure 12E**), including T cells, NK cells, monocytes, dendritic cells (DC), macrophages, B cells, epithelial cells, tissue stem cells, and endothelial cells. In conjunction with the visualization of the PSMD14 expression pattern (**Figure 12F**), we observed that PSMD14 exhibited high expression levels in macrophages, DCs, tissue stem cells, endothelial cells, and epithelial cells (**Figure 12G**). It is noteworthy that, within the tumor group, PSMD14 expression was present in DCs, tissue stem cells, endothelial cells, and epithelial cells, whereas, in the control group, its expression was confined to DCs and macrophages (**Figure 12H**).

### Protein expression of PSMD14 in human tissues

**Figure 13:**
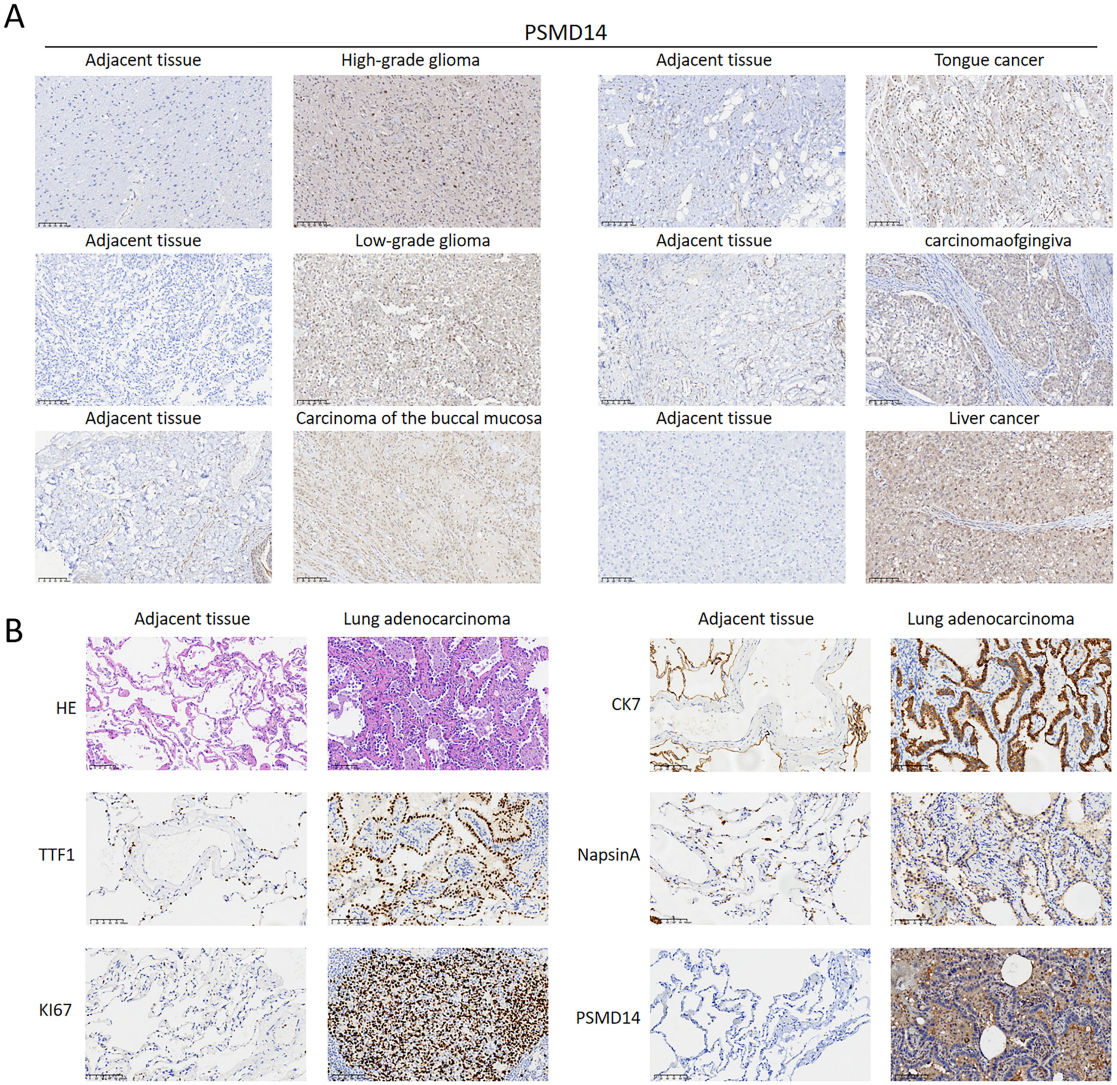
PSMD14 expression levels in tissue samples **(A)** The expression levels of the PSMD14 protein in various human cancer tissues. **(B)** Validation of PSMD14 expression in clinical samples collected from lung cancer patients.

Through our immunohistochemical examination of human pan-cancer specimens, we found a strong correlation between elevated levels of PSMD14 and the progression of diverse malignancies, especially in high-grade gliomas, low-grade gliomas, carcinoma of the buccal mucosa, carcinomaofgingiva, tongue cancer, and liver cancer (**Figure 13A**). This observation indicates that PSMD14, functioning as a deubiquitinase, may be integral to tumor advancement. Additionally, a notable decrease in PSMD14 expression was observed in adjacent non-cancerous tissues, highlighting its potential utility as a tumor biomarker with clinical relevance. In our investigation of LUAD, our findings demonstrated significantly increased expression levels of PSMD14, TTF1, KI67, CK7, and NapsinA in tumor tissues when compared to adjacent non-cancerous tissues, further supporting the role of PSMD14 across various cancer types (**Figure 13B**). In summary, the elevated expression of PSMD14 is not only linked to the initiation and progression of multiple cancers but also holds promise as a crucial biomarker.

### Validation of the prognostic model for PSMD14 in the GEO database

**Figure 14:**
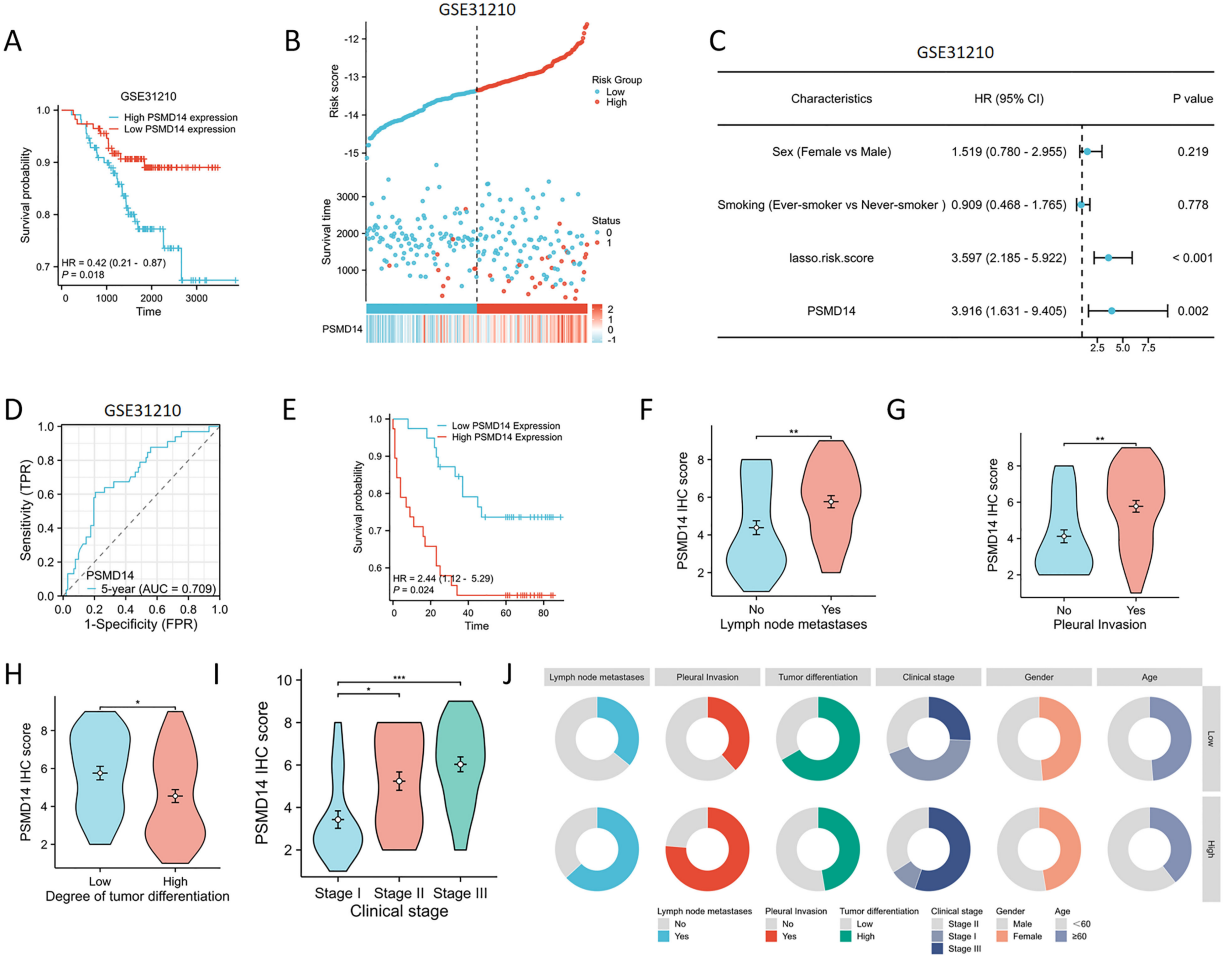
Validating the PSMD14 prognostic model with external datasets and a cohort of 77 patients with LUAD **(A)** High PSMD14 expression was associated with poor OS in LUAD in GSE31210. **(B)** Proportion of mortality as risk score values escalated within low and high-risk groups in GSE31210. **(C)** Time-dependent survival ROC curves were constructed to predict 5-year survival rate in LUAD patients in GSE31210. **(D)** Univariate Cox regression analyses were employed to examine the relationships between OS and various clinical variables in GSE31210. **(E)** KM plot of the survival outcomes of patients with LUAD, categorized by high or low PSMD14 expression levels. **(F–I)** The elevated level of PSMD14 expression was correlated with lymph node metastases, pleural invasion, low differentiation, and advanced clinical stage. **(J)** The circular diagram depicts the correlation between the expression levels of PSMD14 and a range of clinical pathological characteristics linked to LUAD. *, *p*<0.05; **, *p*<0.01; ***, *p*<0.001.

To assess the reliability of the risk model developed from the previous TCGA dataset, we utilized the GSE31210 dataset from the GEO for external validation purposes. The KM analysis revealed a significant association between elevated expression levels of PSMD14 and negative clinical outcomes (**Figure 14A**). In addition, we analyzed the expression profiles of PSMD14 in conjunction with risk scores, survival durations, and the distribution of survival statuses within the GEO dataset. The analytical results demonstrated that PSMD14 expression levels in the high-risk group were significantly higher than those in the low-risk group, correlating with poorer prognoses (**Figure 14B**). This finding is consistent with the data derived from the TCGA training set. To evaluate the prognostic model’s precision, we employed ROC curve analysis, with the GSE31210 cohort yielding an AUC value of 0.709 over a five-year period (**Figure 14C**). Furthermore, a univariate Cox regression analysis confirmed that PSMD14 expression functions as an independent prognostic factor for LUAD patients within the GSE31210 cohort (**Figure 14D**). The results highlight the significant prognostic role of PSMD14 in LUAD.

### Association of PSMD14 expression with prognostic clinical factors in patients with LUAD

We collected clinical data from a cohort of 77 patients to assess the prognostic relevance of the PSMD14 protein, along with its association with clinical and pathological characteristics in lung cancer. Among patients diagnosed with LUAD, those exhibiting diminished levels of PSMD14 showed markedly improved survival outcomes in comparison to individuals with elevated PSMD14 expression (**Figure 14E**). Additionally, we examined the correlation between PSMD14 expression and several clinical parameters. Increased PSMD14 levels were associated with lymph node metastasis, pleural invasion, poor tumor differentiation, and advanced clinical stages (**Figures 14F–J**, **Table 3**). These results suggest that PSMD14 possesses significant prognostic implications in LUAD and may serve as a promising therapeutic target for treatment strategies.

**Table 3:** PSMD14 expression is linked to the clinicopathological features in a group of 77 LUAD patients.

Characteristics	Low PSMD14 expression	High PSMD14 expression	P value
n	39	38	
Lymph node metastases, n (%)			0.017
No	25 (32.5%)	14 (18.2%)	
Yes	14 (18.2%)	24 (31.2%)	
Tumor differentiation, n (%)			0.087
Low	13 (16.9%)	20 (26%)	
High	26 (33.8%)	18 (23.4%)	
Clinical stage, n (%)			0.003
Stage II	12 (15.6%)	13 (16.9%)	
Stage I	17 (22.1%)	4 (5.2%)	
Stage III	10 (13%)	21 (27.3%)	
Gender, n (%)			0.906
Male	20 (26%)	20 (26%)	
Female	19 (24.7%)	18 (23.4%)	
Age, n (%)			0.414
<60	20 (26%)	23 (29.9%)	
≥60	19 (24.7%)	15 (19.5%)	

## Discussion

Cancer represents a significant risk to human health. Despite extensive efforts aimed at enhancing the methods of cancer diagnosis and treatment, the five-year overall survival rates for the majority of cancer types remain alarmingly low. Consequently, there is an immediate necessity for innovative diagnostic and therapeutic strategies. The TCGA database has performed an analysis of multi-omics data spanning 33 prevalent cancer types, thereby presenting unparalleled opportunities to investigate gene functionalities across various cancers (35, 36). This research specifically centers on LUAD, a prevalent and highly aggressive subtype of non-small cell lung cancer (NSCLC), characterized by a rising incidence and unfavorable prognosis (37). The intricate nature of LUAD is intimately linked to the tumor microenvironment and its interactions with the immune system (38, 39). The exploration of PSMD14’s function in LUAD is motivated by its possible relevance in tumor advancement and immune evasion. Contemporary methodologies in cancer research have predominantly concentrated on diverse mechanisms of immune escape; however, the precise relationship between PSMD14 expression and tumor immune infiltration has yet to be adequately examined.

In this investigation, a thorough methodology is adopted, incorporating RNA-sequencing data analysis, single-cell RNA-sequencing analysis, assessments of immune infiltration, survival analyses, and gene enrichment analyses to clarify the complex role of PSMD14 in LUAD. The results reveal a notable association between increased PSMD14 expression and adverse prognosis, highlighting its potential to serve as a novel biomarker for lung adenocarcinoma. The implications of these findings extend beyond simple correlation, indicating that PSMD14 may affect the tumor microenvironment and immune responses, thereby enhancing the understanding of the pathogenesis of LUAD while opening new pathways for therapeutic interventions. Furthermore, the results imply that PSMD14 may aid in tumor initiation and progression through the regulation of genes related to the cell cycle and its interactions with immune cells. This research not only addresses a crucial gap in the current literature but also sets the stage for subsequent studies that aim to utilize PSMD14 in clinical contexts.

This investigation underscores the pivotal function of PSMD14 in LUAD, particularly highlighting its prospective involvement in the regulation of cellular proliferation, signaling pathways, and immune responses. The results indicate a substantial correlation between elevated levels of PSMD14 expression and tumor progression, as well as adverse patient prognosis, thereby offering fresh perspectives on its viability as a biomarker. Specifically, the increased expression of PSMD14 is intricately associated with the modulation of genes related to the cell cycle, implying its potential contribution to the initiation and advancement of tumors through its influence on cell cycle dynamics. Earlier research has recognized PSMD14 as a deubiquitinase that facilitates tumor progression across multiple cancer forms (18, 40). Consequently, exploring the specific mechanisms through which PSMD14 influences the cell cycle is essential for comprehending its function in LUAD.

Moreover, the interplay between PSMD14 and other tumor-associated genes may further clarify its significance in cancer biology. For instance, the positive correlation observed between PSMD14 and other members of the JAMM family, such as EIF3H and PSMD7, suggests the possibility of a synergistic mechanism governing the regulation of oncogenic pathways. A comprehensive understanding of these interactions could unveil the molecular underpinnings of LUAD and highlight prospective therapeutic targets. Additionally, KEGG pathway analysis has demonstrated that PSMD14 is significantly enriched in signaling pathways associated with the cell cycle and nicotine dependence, indicating its integral role in a multitude of biological processes. Notably, the modulation of cell cycle-related signaling pathways could introduce new therapeutic targets for cancer intervention, which is vital for addressing tumor progression (41, 42). The involvement of PSMD14 in nicotine addiction pathways provokes compelling inquiries regarding its influence on the tumor microenvironment. Given nicotine’s established impact on tumor progression and immune responses, PSMD14 may act as a crucial mediator of these effects. Consequently, targeting PSMD14 could represent an innovative strategy for drug development aimed at disrupting these signaling networks, potentially enhancing treatment outcomes for patients with LUAD. Prior studies have proposed that focusing on PSMD14 and its related signaling pathways could furnish a theoretical basis for the creation of novel anticancer therapies (8, 17). Future investigations should emphasize elucidating the role of PSMD14 within various tumor microenvironments and its potential roles in alternative signaling pathways, particularly within the immune microenvironment, as this could reveal new avenues for cancer immunotherapy.

Our investigation into immune cell infiltration underscores the pivotal role of PSMD14 within the tumor microenvironment of LUAD. Importantly, the expression levels of PSMD14 demonstrate a positive correlation with the infiltration of Th2 and Tgd cells, while showing a negative correlation with eosinophils, natural killer (NK) cells, and mast cells. This distinctive pattern of infiltration implies that PSMD14 may be instrumental in modulating the immune landscape of LUAD, potentially affecting tumor progression and mechanisms of immune evasion. Th2 cells, recognized for their role in enhancing humoral immunity and mediating allergic reactions, may signify a transition towards an immune-suppressive environment that favors tumor proliferation (43–45). The presence of Tgd cells, which can possess both pro-inflammatory and regulatory characteristics, adds further complexity to the immune response observed within the tumor (46, 47). Conversely, the negative association with cytotoxic immune cells, like NK cells, highlights a potential immune evasion mechanism, in which the tumor microenvironment may actively inhibit effective anti-tumor responses (48, 49). A comprehensive understanding of the specific interactions between these immune cell types and PSMD14 could yield critical insights into the immune evasion tactics utilized in LUAD.

Moreover, the analysis of single-cell RNA sequencing indicates a notable reduction in the populations of T cells, NK cells, and monocytes within the tumor microenvironment, accompanied by an increase in B cells. This alteration implies a likely immunosuppressive environment that may hinder therapeutic efficacy. The decline in cytotoxic T cells and NK cells, which are vital for tumor detection and eradication, raises significant concerns regarding the effectiveness of existing treatment modalities (50–52). The observed increase in B cells, often linked to tumor-enhancing processes, adds further complexity to the immune landscape, underscoring the necessity for targeted approaches aimed at reestablishing robust anti-tumor immunity. Furthermore, clustering analysis executed via the Seurat package delineated 18 distinct cell clusters, which include a variety of immune cell types such as T cells, NK cells, and macrophages. A comprehensive characterization of the immune cell composition in the tumor microenvironment is crucial for elucidating the intricate interactions that affect tumor dynamics and patient prognoses. Recognizing specific immune cell subsets and their functional states could guide the formulation of innovative immunotherapeutic strategies customized to the distinct immune profiles of LUAD patients (53, 54). Additionally, our single-cell RNA sequencing analysis uncovered multiple immune cell types, including dendritic cells and macrophages, with varying expression levels of PSMD14. In the tumor cohort, heightened PSMD14 expression was observed in DCs, tissue stem cells, endothelial cells, and epithelial cells, contrasting sharply with the limited expression noted in the control group, which emphasizes the potential role of PSMD14 in modulating the immune microenvironment. Dendritic cells, which are essential for antigen presentation and T-cell activation, may significantly affect the overall immune response to tumors through their engagement with PSMD14. These results highlight the necessity for further exploration into the influence of PSMD14 on the immune environment and its prospective implications for therapeutic interventions.

In the present investigation, we leveraged a publicly accessible database to analyze the mRNA expression levels and prognostic relevance of PSMD14 in various cancer types. Our results demonstrate that PSMD14 displays significantly altered expression across a spectrum of malignancies, including lung, liver, pancreas, head, and neck cancers, as well as mesothelioma. The role of PSMD14 is heterogeneous among different cancer types, with elevated expression levels correlating with diminished OS in patients with LUAD, LIHC, PAAD, and HNSC. While the expression of PSMD14 is associated with particular cancer types, it predominantly correlates with advanced stages of disease, especially in LUAD. These findings imply that the expression and prognostic relevance of PSMD14 are notably specific to individual cancers, necessitating further exploration of its distinct functions across different malignancies. Furthermore, we corroborated PSMD14 expression in clinical specimens from LUAD, LIHC, HNSC, and glioma, substantiating its upregulation during the initiation and progression of various tumors. PSMD14 may affect tumor biology by influencing intracellular signaling pathways, facilitating tumor cell proliferation, and hindering apoptosis. Likewise, the increased expression of PSMD14 in head and neck squamous cell carcinoma, glioma, and liver cancer indicates its potential as both a biomarker and a therapeutic target. In summary, the expression patterns and functional implications of PSMD14 across various malignancies lay the groundwork for future research, with subsequent studies anticipated to clarify its specific mechanisms in tumorigenesis and investigate its viability as a target for therapeutic strategies.

In summary, the present study underscores the critical involvement of PSMD14 in lung adenocarcinoma, especially regarding its capacity to influence cellular proliferation, immune responses, and the tumor microenvironment. The results presented herein open up novel avenues for the advancement of biomarkers and targeted treatment options for lung adenocarcinoma. Subsequent investigations should prioritize the clinical implications of PSMD14 and its viability as a therapeutic target within the realm of immunotherapy, to provide enhanced treatment modalities for patients diagnosed with lung adenocarcinoma.

## Data Availability

The original contributions presented in the study are included in the article/supplementary material. Further inquiries can be directed to the corresponding authors.
